# Effect of Different High-Temperature Heating Methods on the Glycation Reaction and Advanced Glycation Reaction Products of *β*-Lactoglobulin

**DOI:** 10.3390/foods14213722

**Published:** 2025-10-30

**Authors:** Xueying Zhang, Qiannan Jiang, Jiaojiao Liu, Hui Wang, Haiyan Lu, Danting Liu, Pingwei Wen, Zongcai Tu, Yueming Hu

**Affiliations:** 1 State Key Laboratory of Food Science and Resources, Nanchang University, Nanchang 330047, China; 2 Nanchang University-Jinggangshan Green Food New Quality Productivity Transformation Center, Ji’an 343016, China; 3 City College of Huizhou, Huizhou 516000, China; 4 National R & D Center of Freshwater Fish Processing, and Engineering Research Center of Freshwater Fish High-Value Utilization of Jiangxi Province, Jiangxi Normal University, Nanchang 330022, China

**Keywords:** *β*-lactoglobulin, glycation, products, UPLC-HCD-MS/MS

## Abstract

*β*-lactoglobulin (*β*-Lg), the major whey protein containing nine lysine residues, serves as an ideal model for studying protein glycation and thermal processing safety in dairy products. This study systematically compared three different high-temperature treatment methods, namely superheated steam (SS), hot air (HA), and oil bath (OB), to investigate their effects on the spatial conformation and glycation product formation of proteins in the *β*-Lg-glucose system. The results show that compared with OB and HA, SS has a lower degree of glycation, lower consumption of free amino groups, and less unfolding of the protein’s three-dimensional structure. It leads to a lower proportion of α-helix transformation into *β*-sheet and random coil in the protein. SS resulted in the least browning and produced less 5-hydroxymethylfurfural, pentosidine, fluorescent advanced glycation end products, and melanogenin, yet produced the highest amount of Carboxymethyllysine. Mass spectrometry analysis shows that lysine residues were the primary glycation sites. Therefore, this work provides molecular-level insights into how different heating techniques modulate protein glycation and structural stability, supporting the potential of superheated steam as a gentler alternative to control glycation for *β*-Lg in food thermal processing.

## 1. Introduction

Milk is a critical component of daily diets and food processing. Whey, as one of the major constituents of milk protein, is essential for optimizing its application in production and processing. *β*-Lactoglobulin (*β*-Lg) is a significant protein in bovine whey, comprising approximately 50–55% of the total whey protein mass [1]. It has been extensively employed in the food industry and occupies a significant position among food industry constituents due to its exceptional nutritional value and functional characteristics.

Glycation modification is a frequently employed protein chemical modification method, which refers to the covalent binding between the carbonyl group of reducing sugars and the free amino groups of proteins [2]. The glycation reaction is a multifaceted chemical process that is influenced by a variety of factors, such as temperature, time, and reaction conditions. The products formed as a result of the reaction are structurally diverse and differ based on the types of sugars and proteins that are involved. In comparison to the conventional long-duration, low-temperature, moist glycation method, the short-duration, high-temperature, dry glycation method is more effective, and time-efficient [3]. Glycation reactions in food are more likely to occur at higher temperatures, according to research findings [4]. In recent years, glycation modification has been used more frequently to enhance the physicochemical and functional properties of food proteins, rendering it a more natural and efficient method than other chemical methods. Food products can acquire distinctive color, flavor, texture, and consistency through glycation.

The body’s tissue cells can be damaged by the advanced glycation end products (AGEs) that result from late-stage glycation, which can contribute to accelerated aging and the occurrence or progression of many chronic diseases [5]. This has become an important focus in glycation research. Additionally, the organism may experience substantial adverse effects as a result of the accumulation of AGEs in the body, which can initiate a series of inflammatory responses and oxidative stress reactions. Therefore, AGEs in food have a significant toxic effect on the human body, and reducing the levels of AGEs in the body is of great importance for human health.

The impact of various high-temperature heat treatment techniques on protein glycation reactions and their products in food remains unknown, despite the increasing use of these techniques in both the food industry and everyday life, including baking, microwaving, frying, and superheated steam. This research will assist in the development of guidelines for the appropriate implementation of high-temperature thermal treatment techniques in the food processing and cooking industries.

This research directly investigates glycation in bovine milk *β*-lactoglobulin (*β*-Lg). The study uses *β*-Lg in a model system with glucose to simulate how this specific milk protein undergoes glycation under different high-temperature processing methods (superheated steam, oil bath, hot air). It aims to investigate the mechanisms underlying the effects of three high-temperature treatment methods, namely superheated steam, oil bath, and hot air, on the glycation products of *β*-Lg. The extent of the reaction between *β*-Lg and glucose during high-temperature treatments was investigated by monitoring the degree of browning, melanoidin formation (MeH), and advanced glycation end-product content. The conformational changes in glycation-modified *β*-Lg were monitored using conventional spectroscopic techniques. The glycation sites and the average degree of substitution per peptide molecule (DSP) during the glycation process were characterized at the molecular level using high-performance liquid chromatography coupled with high-energy collision-induced dissociation tandem mass spectrometry (HPLC-HCD-MS/MS). Therefore, the findings are crucial for understanding and optimizing thermal processes in the dairy industry to potentially enhance flavors and functional properties of milk proteins while mitigating the formation of unhealthy AGEs in bovine milk-based products.

## 2. Materials and Methods

### 2.1. Materials and Chemicals

*β*-Lg (A-5503, Grade V), leucine, Coomassie brilliant blue, sodium dodecyl sulfate (SDS), O-Phthaldialdehyde (OPA), and protein marker were received from Beijing Solarbio Science & Technology Co., Ltd. (Beijing, China). Acetic acid, trichloroacetic acid, methanol, and sodium tetraborate were of analytical reagent grade and purchased from Tianjing Damao Chemical Reagent Factory (Tianjin, China). The rest of the reagents were of analytical reagent grade.

### 2.2. Sample Preparation

Preparation of glycated *β*-Lg occurred as follows. Dry-state glycation was used to prepare glucose-*β*-Lg conjugation. *β*-Lg was mixed with glucose in a ratio of 1:1 (*w*/*w*) and a final concentration of 10 mg/mL. The time-temperature parameters were selected to simulate High-Temperature Short-Time (HTST) industrial processes while enabling a systematic comparison of the three distinct heating methods. After 48 h of freeze-drying, the mixture was treated in hot air, oil bath, and superheated steam for 2, 4, and 6 min, respectively. All samples were stored at −20 °C before use. Unprocessed *β*-Lg was designated as N-*β*-Lg (blank control). The *β*-LG treated with hot air for 2, 4 and 6 min were named HA-2, HA-4 and HA-6. After oil bath treatment, they were named OB-2, OB-4 and OB-6. After superheated steam treatment, they were named SS-2, SS-4 and SS-6. The processing temperature of HA, OB and SS was 130 °C. To quantify various indicators, all *β*-Lg samples were dissolved in ultrapure water.

### 2.3. Determination of Free Amino Group Content and Kinetic Model

Referring to the method of Yang et al. [6], a total of 0.20 g of OPA and 0.22 g of DTT were dissolved in 5 mL of 95% ethanol. A total of 9.525 g of borax and 0.25 g of SDS were dissolved in 100 mL of ultrapure water. Then, we mixed the two solutions and transferred them to a volumetric flask, and diluted them with ultrapure water to a final volume of 250 mL. This solution is called OPA reagent. A total of 0.1 mL of *β*-Lg sample (1 mg/mL) and 2 mL of freshly prepared OPA reagent were mixed and then incubated in the dark at 37 °C for 2 min. The absorbance of the mixture at 340 nm was immediately measured using the U-2910 spectrophotometer (Hitachi, Tokyo, Japan). With L-lysine concentrations (0.025–0.50 mg/mL) were used to generate the standard curve. The measurements of all samples were performed in triplicate. The reversible first-order reaction kinetics model in the study with Li et al. [7] was adopted to further describe the consumption of free amino groups. The kinetic equations are then shown in Formula (1):

(1) $$[P]=[P{]}_{eq}+([P{]}_{0}+[P{]}_{eq})e^{-kt}$$

where k is the apparent rate constant and [P] is the content of free amino, respectively, at time t. The value of [P] at time zero is [P]_0_. The values of [P] at equilibrium are [P]_eq_, respectively. In the present work, measured values of [P] versus time were used to obtain the fitted parameter values [P]_eq_, and k.

### 2.4. Sodium Dodecyl Sulfate–Polyacrylamide Gel Electrophoresis (SDS-PAGE)

Refer to the method of Zhang et al. [8]. The volume ratio of 16.5% separating glue, 10% sandwich glue and 4% concentrated glue was 4:1.5:1. Take 9 μL (1 mg/mL) of sample, add 3 μL of loading buffer, mix, centrifuge, and boil in the water bath for 5 min. Ultra low molecular weight marker (3.3 KD–31.0 KD) was used as control. Run the electrophoresis at 30 V for 1–2 h, and adjust it to 100 V until the leading edge reaches the upper edge of the separating gel. After electrophoresis, the gel was fixed in the fixing solution for 20 min and then stained with Coomassie brilliant blue for 20 min. Finally, place the decolorizing solution for about 24 h until the bands were clear. Imaging devices were used to record the scanning process (ChemiDoc, Bio-Rad, Singapore).

### 2.5. Measurement of Fluorescence Spectroscopy and UV Absorption Spectroscopy

Referring to the method of Liao et al. [9], the sample concentration was 1 mg/mL. The intrinsic emission fluorescence spectra parameters were specified as follows: excitation wavelength of 280 nm, emission wavelength scan range of 300–400 nm, slit width of 5 nm, and scanning speed of 1200 nm/min. The lyophilized samples were diluted to 1 mg/mL with PBS (10 mM, pH 7.4). A full-range UV spectrometer was used to scan the samples at a scanning rate of 200 nm/min and an aperture width of 1.50 nm, with an effective wavelength range of 250–350 nm.

### 2.6. Far-UV CD Spectroscopy

The Far-UV CD spectroscopy of *β*-Lg samples was conducted using a French Bio-Logic SAS spectropolarimeter (MOS-450; Claix, France). The parameters of the Far-UV CD spectra were as follows: a path length of 1.0 mm, a scan rate of 100 nm/min, and a wavelength range of 190–250 nm. Subsequently, the contents of the secondary structures (*α*-helix, *β*-sheet, *β*-turn, and random coil) were obtained using DichroWeb (http://dichroweb.cryst.bbk.ac.uk/html/home.shtml, accessed on 14 June 2024).

### 2.7. Determination of Degree of Browning (DOB)

Referring to the method of Jung et al. [10] to determine different heating methods and heating times, the absorbance values of the *β*-Lg-glucose system were measured at 294 nm and 420 nm.

### 2.8. Determination of Main Products

#### 2.8.1. Determination of 5-Hydroxymethylfurfural (5-HMF) Content

Referring to the method of Jiang et al. [11], high-performance liquid chromatography (HPLC) was employed to determine the content of 5-HMF in the sample. The content of 5-HMF in the samples was determined by high-performance liquid chromatography (HPLC). Mix a sample of 20 mg/mL, ultrapure water, and 6 M HCl in a ratio of 1:8:1 (*v*:*v*:*v*), seal the mixture, and boil it for 15 min. Centrifuge at 4000 rpm for 20 min and filter using a 0.22 μm aqueous membrane. Use an Agilent C_18_ column (5 μm, 4.6 × 150 mm). The mobile phase consists of methanol and water in a 30:70 (*v*:*v*) ratio. The flow rate is 0.4 mL/min, and the detection wavelength is 285 nm. Simultaneously, detect the HMF standard at concentrations of 5~25 μg/mL. The retention time and peak area from the analysis results were used to construct a standard curve, which was then employed to ascertain the 5-HMF content in the samples.

#### 2.8.2. Determination of Pentosidine Content

Refer to the method of Lima et al. [12]. The content of pentosidine in the protein sample is typically determined by diluting the sample to 2 mg/mL. Fluorescence intensity readings of the sample are obtained using a fluorescence spectrophotometer (F-7000, Hitachi, Tokyo, Japan). The excitation wavelength used for the measurement is set to 335 nm, while the emission wavelength is set to 385 nm. The fluorescence intensity measured at these wavelengths indicates the content of pentosidine in the samples.

#### 2.8.3. Determination of Fluorescent AGEs (F-AGEs) Content

Refer to the method of Fang et al. [13]. The samples were diluted to a concentration of 0.1 mg/mL with PBS (10 mM, pH 7.4). The fluorescence intensity of each sample was measured at an excitation wavelength of 370 nm and an emission wavelength of 440 nm using a Hitachi F-7000 fluorescence spectrophotometer (Hitachi, Japan). These parameters can be used to characterize the content of fluorescent advanced glycation end products.

#### 2.8.4. Determination of Melanoidins (MLD) Content

Refer to the method of Zhang et al. [14]. The sample concentration is 20 mg/mL. Measure the absorbance of the sample solution at a wavelength of 470 nm using the following Formula (2):

(2) $$C\;=\;\frac{A\;\times \;V\;\times \;1000}{e\;\times \;b},$$

Among them, the unit of C is mmol/L; A represents the detected absorbance; V represents the sample volume (in mL); e is the molar extinction coefficient, which is 282 L/(mol·cm); and b is the thickness of the colorimetric cuvette (in cm).

#### 2.8.5. Determination of Carboxymethyl Lysine (CML) Content

Refer to the method of Tauer et al. [15]. CML levels in samples were determined using an enzyme-linked immunosorbent assay (ELISA) competitive method. A solid-phase antibody was generated by coating purified CML antibodies onto microplate wells. In order to compete for binding, CML and horseradish peroxidase (HRP) labeled CML antigens were introduced into the monoclonal antibody-coated wells. Substrate TMB was added for color development after a thorough rinsing. The content of CML in the samples and the intensity of the sample pigment exhibited a negative correlation. The ELISA reader was used to measure the absorbance (OD value) at a wavelength of 450 nm, and the CML content in the samples was determined using a standard curve.

### 2.9. HPLC-HCD-MS/MS

Based on the research of Yang et al. [16], a study was conducted to investigate the glycation sites and the average degree of substitution per peptide (DSP) molecule of *β*-Lg-Glu. The SS-4, OB-4, and HA-4 samples were diluted and subjected to pepsin digestion in a 1:1 (*w*/*w*) ratio in HCl solution at pH 2.2, subsequently incubated at 4 °C for 10 min. The effluents from the samples were injected into an LTQ-Orbitrap Fusion mass spectrometer for mass spectrometry analysis. Positive ions detected in the precursor ion scan were fragmented through high-energy C-trap dissociation (HCD) to obtain fragment ions.

To further compare the extent of glycation for each peptide, the DSP of *β*-Lg was calculated using the following Formula (3):

(3) $$\mathrm{DSP}\;=\;\frac{\sum_{i=0}^{n}{i\;\times \;I}_{\left(\mathrm{peptide}+i\times \mathrm{suger}\right)}}{\sum_{i=0}^{n}I_{\left(\mathrm{peptide}+i\times \mathrm{suger}\right)}},$$

Among them, I and i, respectively, represent the product of the strength of each glycosylated *β*-Lg peptide and the number of glucose units attached to the corresponding peptide. ΣI represents the total strength of all glycosylated *β*-Lg peptides.

### 2.10. Statistical Analysis

All experiments were performed in triplicate, and the mean ± standard deviation was used to represent all data. SPSS 17.0 for Windows (SPSS Co., Chicago, IL, USA) was used to analyze the data variance, and *p* < 0.05 was considered statistically significance. The Origin 2019 (OriginLab Co.) was employed for graphing.

## 3. Results and Discussion

### 3.1. Determination of Free Amino Group Content and Kinetic Analysis

**Figure 1:**
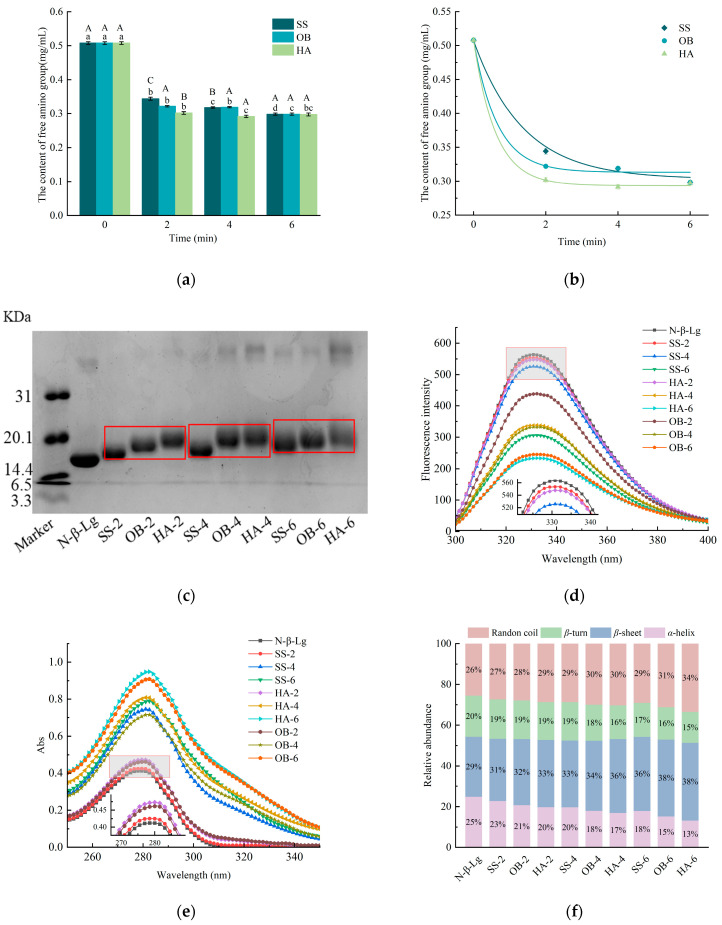
The free amino content (**a**), kinetic fitting curves (**b**), SDS-PAGE profiles (**c**), intrinsic fluorescence spectra (**d**), UV spectra (**e**), and circular dichroism spectra (**f**) of glycated *β*-Lg treated with different high-temperature heating methods. N-*β*-Lg represents untreated *β*-Lg. HA represents hot air treatment, OB represents oil bath treatment, and SS represents superheated steam treatment. The numbers 2, 4, and 6 represent heating for 2 min, 4 min, and 6 min, respectively. Lowercase letters a–d denote significant differences (*p* < 0.05) among samples subjected to the same heat treatment but with different durations. Uppercase letters A–C represent significant differences (*p* < 0.05) among samples subjected to the same duration but with different heat treatments.

The amino groups of proteins will form covalent bonds with the carbonyl groups of reducing sugars through glycation reactions, which will consume free amino groups and lead to a decrease in the content of free amino groups [17]. The content of free amino groups can be used to reflect the degree of Maillard reaction at the macro level [18]. The temperature at 130 °C changes in the free amino group content of *β*-Lg after three types of high-temperature glycation treatments are shown in Figure 1a. Compared to N-*β*-Lg, after being treated with three different heating methods, the free amino acid content of *β*-Lg has significantly decreased (*p* < 0.05), indicating that *β*-Lg and glucose undergo covalent binding as the dry heat reaction progresses. During the entire reaction process, the free amino acid content of the protein sample treated with hot air decreased the fastest, while the free amino acid content of the protein treated with superheated steam decreased the slowest and ultimately reached the same stable state. For 9 samples with different processing methods and reaction times, their free amino content was in descending order of HA-6 (0.291), OB-6 (0.295), SS-6 (0.297), HA-4 (0.298), HA-2 (0.302), OB-4 (0.319), SS-4 (0.318), OB-2 (0.322), and SS-2 (0.344). At the same time, this order can also characterize the degree of activity of proteins undergoing glycation reactions under dry heat conditions. Glucose rapidly breaks down to produce more carbonyl groups, making it easier for glucose to react with proteins. Overheated steam causes a layer of water film to adhere to the interface, resulting in slow glucose degradation and low carbonyl content [19], reducing the possibility of covalent binding between proteins and sugars, thus reducing the loss of amino groups.

Based on the content of free amino groups, a kinetic analysis was performed to quantitatively compare the glycation rates induced by the different heating methods. Three kinetic fitting curves (SS, OB, HA) starting from 0.507 mg/mL at time zero and asymptotically approaching their respective equilibrium concentrations were shown in Figure 1b. The HA curve would be the steepest, followed by OB, and then is SS. As shown in Table 1, the fitted parameter values were k = 0.725 ± 0.211 min^−1^ and [P]_eq_ = 0.303 ± 0.011 mg/mL for SS, k = 1.520 ± 0.771 min^−1^ and [P]_eq_ = 0.313 ± 0.010 mg/mL for OB, and k = 1.645 ± 0.336 min^−1^ and [P]_eq_ = 0.294 ± 0.003 mg/mL for HA. This progression of rate constants quantitatively demonstrates that the intensity of the glycation reaction is highest in HA, intermediate in OB, and mildest in SS. The significantly slower rate observed in SS provides a kinetic explanation for its ‘gentler’ impact. This result suggests that the different heating methods dominate the glycation process mainly by affecting the reaction kinetics rather than altering the final thermodynamic equilibrium since the [P]_eq_ of all three are very close.

### 3.2. SDS-PAGE Analysis

The bands of N-*β*-Lg, SS-2, OB-2, HA-2, SS-4, OB-4, HA-4, SS-6, OB-6, and HA-6 are shown in Figure 1c. Compared to N-*β*-Lg, the bands of the glycated samples shifted upwards, indicating the occurrence of glycation reactions in the samples and the covalent crosslinking between *β*-lactoglobulin and glucose, which can increase the molecular weight of *β*-lactoglobulin. Wang et al. [20] also reported a situation in which *β*-lactoglobulin reacted with arabinose, and its products similarly clustered. In contrast, under the same treatment time, the protein bands of samples modified with SS and OB were significantly lower than those modified with HA, suggesting a lower degree of glycation for proteins treated with SS and OB. Furthermore, dimeric aggregation bands were observed in samples subjected to OB and HA treatments. In connection with the results of the free amino content experiments, it was found that the reason for this phenomenon is the covalent binding of free amino groups to the carbonyl groups on glucose in the hot air-treated protein samples. More sugar grafted onto the protein resulted in a rise in the molecular weight of the protein.

### 3.3. Fluorescence Spectroscopy and UV Absorption Spectroscopy Analysis

The intrinsic fluorescence of proteins is usually generated by tryptophan and tyrosine residues. The intrinsic fluorescence intensity of *β*-Lg samples is shown in Figure 1d. Compared to native *β*-Lg, the fluorescence intensity of glycated *β*-Lg has decreased. The fluorescence intensity values of the samples after glycation significantly decreased from 563.73 (N-*β*-Lg) to 533.73 (SS-2), 439.09 (OB-2), 547.80 (HA-2), 547.68 (SS-4), 331.92 (OB-4), 338.41 (HA-4), 306.07 (SS-6), 245.50 (OB-6), and 233.42 (HA-6) (*p* < 0.05). Among these, the three samples treated with hot air saccharification showed the lowest fluorescence intensity compared to the other two heat treatment methods at the same treatment time, and the fluorescence absorption wavelength slightly shifted from 330 nm to 334 nm. This result indicates that the spatial structure of *β*-Lg changed after glycation modification, which is possibly due to glycation reactions that unfold the protein structure. This structural change exposes Trp residues more in the solvent, thereby reducing fluorescence intensity. This trend is similar to the findings reported by Bian et al. [21].

The protein contains aromatic amino acids that can absorb ultraviolet light and exhibit an absorption peak in the ultraviolet region near 280 nm, which makes it possible to detect the highest absorption peak of *β*-lactoglobulin at 280 nm by ultraviolet spectroscopy. Specifically, hydroxymethylfurfural, a glycation product, also exhibits UV absorption at 284 nm. The tertiary structure of proteins is easily destroyed during glycation, which exposes more aromatic amino acids, leading to an increase in UV absorption intensity. As shown in Figure 1e, the absorption peaks of modified *β*-Lg are higher than those of natural *β*-Lg. The absorption of the samples ranges from 0.412 (N-*β*-Lg significantly increased, (*p* < 0.05) to 0.425 (SS-2), 0.462 (OB-2), 0.473 (HA-2), 0.715 (OB-4), 0.745 (SS-4), 0.786 (SS-6), 0.808 (HA-4), 0.909 (OB-6), and 0.949 (HA-6). This may be due to the modification of reducing sugars, which exposes the residues of tryptophan, tyrosine, and phenylalanine on the surface of *β*-Lg molecules, causing a corresponding increase in absorption peaks. Wang et al. [22] have also reached the same conclusion. The sample with the highest absorbance value among all samples was treated with hot air for 6 min, indicating that under the same reaction conditions, the method of hot air heating has the greatest impact on the conformation of *β*-Lg. In addition, we also observed a phenomenon where the UV absorption wavelength shifted slightly from 279 nm to 282 nm, which may be related to the formation of the intermediate product of the glycation reaction called hydroxymethylfurfural.

### 3.4. Far-UV CD Spectroscopy Analysis

The contents of the secondary structure of *β*-Lg after glycation (*α*-helix, *β*-sheet, *β*-turn, random coil) are shown in Figure 1f. N-*β*-Lg contains 25% *α*-helix, 29% *β*-sheet, 20% *β*-turn, and 26% random coil. The secondary structure changes are not significant in SS-2, while in HA-6, the content of *α*-helix, *β*-sheet, *β*-turn, and random coil reaches 13%, 38%, 15%, and 34%, respectively. It is obvious that the longer the processing time, the greater the change in the secondary structure of the protein. After glycation, the content of *α*-helix significantly decreases (*p* < 0.05), while the content of *β*-sheet and random coil significantly increases (*p* < 0.05). The decrease in *α*-helix content can indicate the unfolding of the protein structure. The results in SDS-PAGE indicate that the modified proteins undergo molecular aggregation, and the aggregation of protein molecules occurs through unfolding, often accompanied by the formation of *β*-sheet [23]. The reduction in *α*-helix content, increase in *β*-sheet formation, and higher content of random coil structures may be attributed to the unfolding of *β*-Lg spatial structure caused by heating reactions and glycation reactions, resulting in a more regularized structure [24,25]. The changes in secondary structure content observed in this study are consistent with those reported in other studies.

### 3.5. Degree of Browning (DOB) Analysis

**Figure 2:**
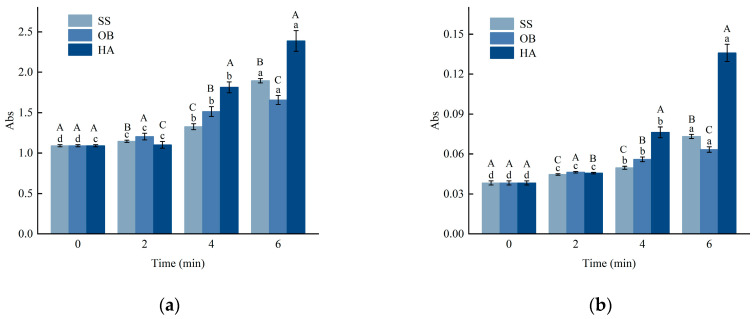
Changes in browning degree (**a**,**b**) of *β*-Lg glucose system under SS, OB, and HA treatment. (**a**,**b**) represent the A_294_ and A_420_ values of the sample treated at 130 °C, respectively. Lowercase letters a–d denote significant differences (*p* < 0.05) among samples subjected to the same heat treatment but with different durations. Uppercase letters A–C represent significant differences (*p* < 0.05) among samples subjected to the same duration but with different heat treatments.

Glycation samples are commonly monitored at 294 nm to assess the initial reaction rate, representing the formation of intermediate products. In comparison, the absorbance value at 420 nm reflects the generation of end products. The absorbance values of A294 and A420 from *β*-lactoglobulin (*β*-Lg)-glucose glycation systems prepared through different high-temperature treatments and reaction times are shown in Figure 2a,b. A294 and A420 showed an increasing trend with longer reaction times (*p* < 0.05). After 2 min of reaction, the absorbance values of the samples did not differ significantly from those of native *β*-Lg, possibly because the response was still in the early stages, and the degree of reaction was low. As the reaction progressed, glucose dehydration led to the accumulation of intermediate products with ultraviolet absorption at 294 nm, resulting in increased absorbance values. This is similar to the findings of previous studies by Jung et al. [10]. The increase in absorbance at 294 nm primarily indicates the accumulation of early-stage Maillard reaction intermediates, particularly carbonylic compounds such as hydroxymethylfurfural (HMF) and its precursors [26], which form during the degradation of Amadori rearrangement products. This measurement serves as a reliable indicator for monitoring the progression of the initial glycation stages in protein-sugar systems. Maillard reaction products, including those generated in this study, inevitably contribute to browning and the development of distinctive flavors, which may be undesirable in products where a neutral color or mild flavor profile is essential. The intense browning and potential bitter notes generated from pronounced glycation, as observed in the hot air (HA) treated samples, could limit their use in light-colored or delicately flavored foods and beverages. With further reaction time, the intermediate products were gradually consumed, and the glycation reaction entered the final stage, leading to increased production of end products. The absorbance intensity of the sample at 6 min indicated that superheated steam (SS) was the milder heat treatment.

### 3.6. Analysis of Glycation Reaction Product Contents

#### 3.6.1. Analysis of 5-HMF Content

**Figure 3:**
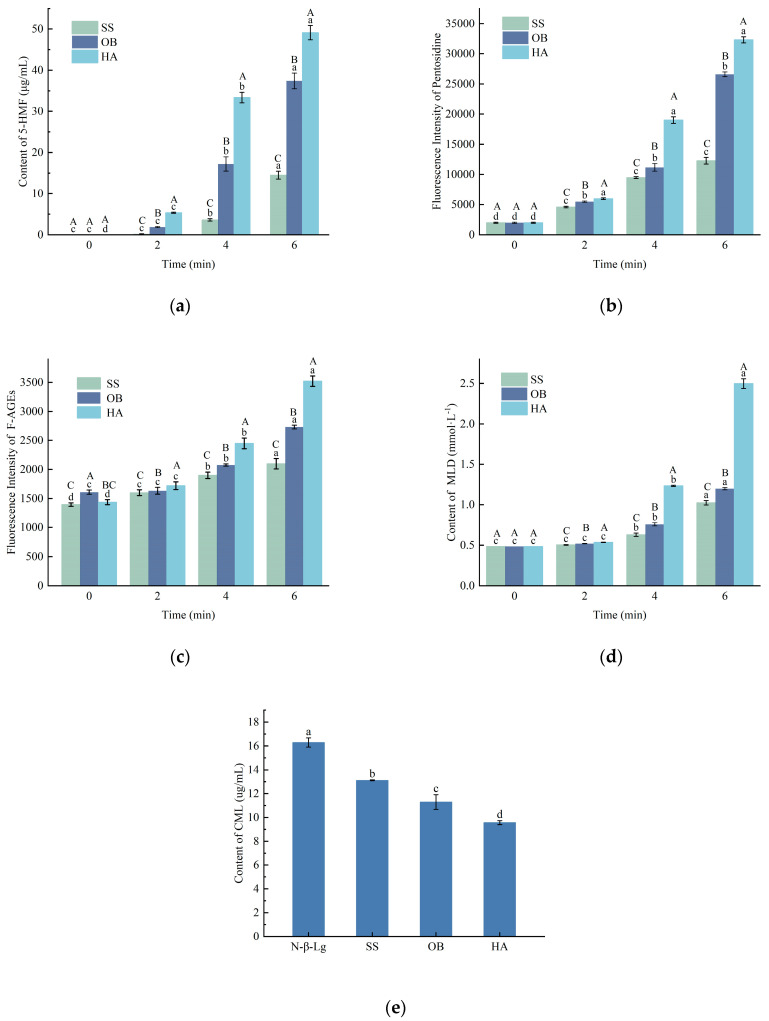
Changes in the content of 5-HMF (**a**), pentosidine (**b**), F-AGEs (**c**), MLD (**d**), and CML (**e**) in the *β*-Lg-glucose system under conditions of SS, OB, and HA treatment. Lowercase letters a–d denote significant differences (*p* < 0.05) among samples subjected to the same heat treatment but with different durations. Uppercase letters A–C represent significant differences (*p* < 0.05) among samples subjected to the same duration but with different heat treatments.

As shown in Figure 3a, at the same heating temperature, the content of 5-HMF increased significantly to varying degrees with increasing heating time from 0 to 6 min. When the heating treatment was conducted at 130 °C for 6 min, the content of 5-HMF reached its highest value (40.10 μg/mL).

5-HMF is an intermediate product of the Maillard reaction and serves as a key indicator for both desirable flavor development and potential formation of advanced glycation end-products. Its monitoring is particularly relevant in dairy processing optimization, where controlling the balance between functional property development and nutritional quality is essential [27]. The content of 5-HMF can reflect the Maillard reaction process induced by heating in the protein-glucose system. The amount of 5-HMF generated in the system is strongly correlated with the degree of heating. The content of 5-HMF increases with increasing heating time. The reason for the above changes may be that the ε-amino group of lysine residues on the protein can participate in the carbonyl Maillard reaction under high-temperature and intense heating conditions, leading to intensified Maillard reaction and rapid production of corresponding 5-HMF. Similarly, researchers such as Sacchetti et al. [28] have found that 5-HMF increases exponentially with heating time.

#### 3.6.2. Analysis of Pentosidine Content

Pentosidine is a fluorescent product generated from protein glycation reactions. As shown in Figure 3b, the trend of pentosidine formation is similar to that of fluorescent AGEs content. There was no significant increase in pentosidine content observed at 2 min, while a substantial increase was observed after 4 min of reaction. Samples treated under higher temperatures exhibited a higher content of pentosidine compared to those treated with overheated steam, and the increasing trend was faster. This may be due to the complex mechanism of end-product formation in protein glycation reactions and the influence of overheated steam conditions on the tendency to form end-products, leading to a lower tendency to form pentosidine in these samples.

#### 3.6.3. Analysis of F-AGEs Content

Fluorescent AGEs are a type of irreversibly harmful glycation end-products formed at a later stage of the Maillard reaction, mainly generated via complex reactions between carbonyl compounds and amino compounds [29]. As shown in Figure 3c, with the extension of reaction time, the content of fluorescent AGEs in the reaction system gradually increases, and the sample treated with hot air has a higher content of fluorescent AGEs than those treated with overheated steam and oil bath, exhibiting a faster growth trend. This indicates that hot air, as a heat transfer medium, has a greater ability to promote formaldehyde condensation and carbonyl compound formation than the other two methods, thus leading to more rapid and increased production of AGEs. In contrast, overheated steam is a mild food thermal processing method [30], which showed the lowest amount of AGEs in the detection system, consistent with the experimental results of Chen et al. [31].

#### 3.6.4. Analysis of MLD Content

During the final stage of the Maillard reaction, samples tend to accumulate and generate brown polymeric compounds known as melanoidins, which are crucial determinants of product color and flavor in thermally processed dairy products and possess various physiological properties such as antioxidant, blood pressure-lowering, and anti-tumor effects [32]. Analysis of the measurement results reveals that the content of melanoidins gradually increases with the prolongation of reaction time, with the highest level observed at 6 min of reaction, as shown in Figure 3d. It can be observed that, under the same reaction time, the content of melanoidins in samples treated under high-temperature steam conditions is significantly higher than those treated under high-temperature baking conditions, indicating that high-temperature steam conditions promote glycation reactions in samples and lead to a higher degree of reaction. However, it can also be seen that at 2 min of response, there is no significant increase in the content of melanoidins in the samples, suggesting that the response is still in the early stage and end products have not yet been formed.

#### 3.6.5. Analysis of CML Content

CML has become a critical marker for monitoring and controlling the nutritional quality and safety of thermal processing. Its accumulation is directly linked to the heat load, and regulating its formation is a key objective in developing milder processing strategies to minimize the formation of potentially harmful compounds. CML is a modified amino acid, and its formation pathway is highly complex. Previous studies have shown [33,34] that in the Maillard reaction, when sugars act as substrates for the formation of CML, the pathway involves the sugar oxidation product, glyoxal, and Amadori rearrangement products. Glyoxal is the major intermediate formed during the automatic oxidation of sugars and reacts with lysine to generate CML, while Amadori rearrangement products are formed through oxidative cleavage and contribute to CML formation. CML has relatively high acid stability, and the determination of CML content can serve as an important indicator to evaluate protein chemical modifications in the Maillard reaction of food systems. In this experiment, the CML content at 4 min was measured for the three heating methods. The results presented in Figure 3e show that the SS sample had the highest CML content (13.11 μg/mL), while the HA sample had the lowest CML content (9.49 μg/mL). This may be attributed to the significant consumption of glucose, the reaction substrate, and the subsequent decrease in reaction rate at the 4 min stage. As a result, the generation rate of CML slows down. Additionally, due to the poor thermal stability of CML, its decomposition rate exceeds its generation rate, leading to a decrease in CML content. Similar trends in CML content were observed in the report by Fu et al. [35].

Although superheated steam minimized most AGEs, its promotion of CML formation warrants attention, as CML is a well-characterized compound with potential health implications. Furthermore, the sensory properties of glycated proteins present significant application barriers. Maillard reaction products, including those generated in this study, inevitably contribute to color changes (browning) and the development of distinctive flavors, which may be undesirable in products where a neutral color or mild flavor profile is essential. The intense browning and potential bitter notes generated from pronounced glycation, as observed in the hot air-treated samples, could limit their use in light-colored or delicately flavored foods and beverages. Therefore, the implementation of these modified ingredients must be carefully evaluated on a case-by-case basis, balancing the targeted functional benefits against the potential for negative sensory impact and the need to minimize dietary AGE intake.

### 3.7. Glycation Sites and DSP Values by Mass Spectrometry

At 2 min, the degree of glycation is still too low to provide reliable site occupancy, while at 6 min, prolonged heating has begun to degrade some early glycation products. Therefore, in order to ensure that the glycation sites detected by LC-MS/MS are both abundant and stable, we chose a 4 min time point.

**Figure 4:**
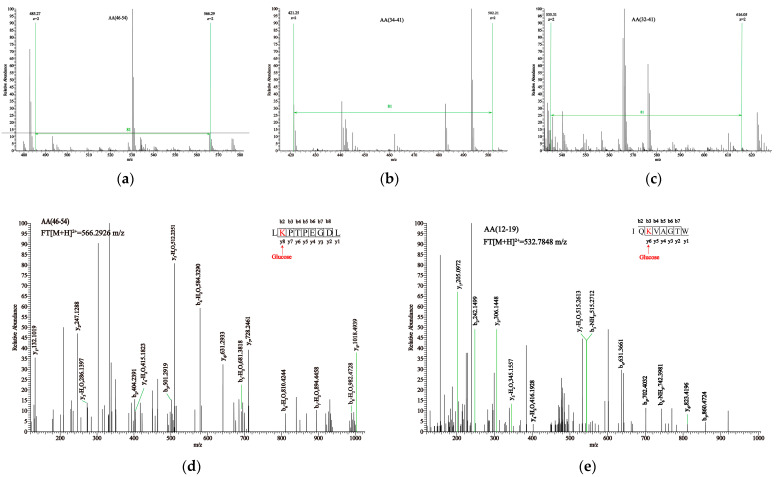
(**a**–**c**) are the primary mass spectra of peptide segments 46–54, 42–54, and 32–31 in SS-4, OB-4, and HA-4 samples, respectively. (**d**,**e**) are the secondary mass spectra of peptide segments 46–54 in the SS sample.

We simultaneously employed HPLC-HCD-MS/MS to determine the glycation sites. Theoretically, if a glucose molecule glycates a peptide, the m/z values of peaks with charges of 1^+^, 2^+^, 3^+^, and 4^+^ will exhibit corresponding mass shifts of 162.0528, 81.0264, 54.0176, and 40.5132, respectively. Based on the changes in mass-to-charge ratio, we performed primary spectrum matching for the glycated samples shown in Figure 4a–c. For example, in the case of SS, the m/z value of the peptide segment aa (46–54) changed from 485.26682^+^ to 566.29262^+^, indicating an 81 m/z shift. Similarly, the aa (122–130) in SS, aa (12–19) and aa (34–41) in OB, and aa (4–13) and aa (32–41) in HA all experienced an 81 m/z shift, changing their respective m/z values from 522.27282^+^, 451.75882^+^, 421.24872^+^, 567.79652^+^, and 535.30502^+^ to 603.29842^+^, 532.78482^+^, 502.27462^+^, 648.82122^+^, and 616.33012^+^.

Primary mass spectrometry is only used to screen potential glycated peptides based on changes in mass. To further identify the glycation sites, we analyzed fragment ions in secondary mass spectrometry, as shown in Figure 4d,e. K47 was identified as a glycation site in the aa (46–54) of SS. Based on this assumption, the theoretical fragment ions of the glycated peptide can be obtained and then matched one by one in the secondary mass spectrometry. The higher the matching degree, the higher the accuracy of the glycation site. 15 fragment ion peaks (b2, b3, b4, b5, b6, b7, b8, y1, y2, y3, y4, y5, y6, y7, and y8) were found in the secondary mass spectrometry of aa (46–54), confirming that K47 in aa (46–54) was modified by glucose. Similarly, in the secondary mass spectrum of aa (12–19), 12 fragment ion peaks (b2, b3, b4, b5, b6, b7, y1, y2, y3, y4, y5, and y6) were found. Therefore, it can be demonstrated that K14 is glycated with glucose.

Using the same identification method, multiple glycation sites were identified in each glycated sample, as shown in Table 2. The results showed that glycation mainly occurred on lysine residues, rather than arginine residues and N-terminal amino acids, which is consistent with previous studies [9]. The identified glycation sites in SS were K8, R14, R40, K47, R169, K70, K83, R124, K100, and K101. Among them, K47 in the aa (46–54) of SS had a DSP of 75.35%, indicating that it was the most active glycation site in SS. K47 (DSP = 86.71%) was confirmed to be the most active glycation site in the aa (42–54) of OB. K14 with a DSP of 96.20% was the most active site in the aa (32–41) of HA.

**Figure 5:**
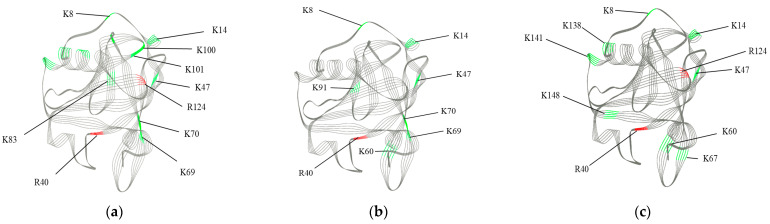
(**a**–**c**) represent the ribbon diagram of samples SS-4, OB-4, and HA-4, respectively. The color-coded as follows: gray indicates the framework of the *β*-Lactoglobulin, red indicates lysine glycation sites, while green indicates arginine glycation sites.

A more intuitive 3D representation is shown in Figure 5. There are 10, 8, and 9 glycation sites present in SS, OB, and HA, respectively. The reason why SS has a low degree of glycation but detects the most glycation sites may be as follows: HA and OB undergo intense glycation reactions, early Schiff bases or Amadori products are converted into cross-linked, cyclized or cleaved products, and their peptide segments disappear from the mass spectrometry signal or exceed the scanning range in m/z. Alternatively, SS treatment may cause the protein structure to stretch, exposing more binding sites on the lysine side chain. However, the higher water content dilutes the carbonyl concentration, resulting in less covalent binding between the amino groups in the protein and the carbonyl groups in glucose.

The increase in glycation sites may be attributed to the thermal reaction that loosens the structure of *β*-Lg, thereby accelerating glycation and exposing more reactive sites [36]. In SS, 3 glycation sites are located in *α*-helical structures, 3 in *β*-sheet structures, 1 in *β*-turn structures, and 5 in random coil structures. In OB, 1 glycation site is located in an *α*-helical structure, 5 in *β*-sheet structures, and 2 in random coil structures. For HA, 2 glycation sites are situated in *α*-helical structures, 4 in *β*-sheet structures, 1 in *β*-turn structures, and 3 in random coil structures. The most active glycation sites in each sample are K47 in SS, K47 in OB, and K8 in HA. Except for K8 in HA, which is located in a random coil structure, all the others are present in *β*-sheet structures. The *β*-sheet structures can make the protein structure more compact, impeding the accessibility of external substances. However, under the treatment of SS and OB, they become glycation sites. This phenomenon may be attributed to the unfolding of protein structures caused by high temperature and the increased permeability, which enhances the probability of collision between *β*-Lg and glucose, and reduces the activation energy required for covalent cross-linking reactions [37].

The finding that specific thermal methods can preferentially unfold *β*-sheet regions to expose residues like K47 means that the functional properties of *β*-Lg, such as emulsification, heat stability, and solubility, can be selectively enhanced by choosing a heating modality that modifies key structural domains in industrial practice. For instance, if a specific *β*-sheet-rich region is known to influence the functions of *β*-Lg, it could select SS to selectively glycate that area, thereby engineering protein ingredients with tailored functionalities [38]. While glycation may improve functionality, the concomitant formation of potentially harmful Advanced Glycation End-products (AGEs) is a concern. The data on the structure-activity relationship of glycation provides a foundation for process control to mitigate AGEs. By understanding the specific unfolding pathways induced by different heating methods, processors can fine-tune parameters (e.g., temperature, time, and heating medium) to achieve a sufficient level of beneficial, surface-level glycation for functionality, while minimizing the deep, extensive unfolding that often leads to the complex cascade of reactions forming hazardous AGEs [39].

## 4. Conclusions

Hot air, oil bath and superheated steam were compared for glycating *β*-lactoglobulin with glucose at 130 °C. The results show that hot air caused the fastest loss of free amino groups, the largest SDS-PAGE shift, the highest UV absorbance, browning and fluorescent AGEs. Characterized by the slowest glycation rate, superheated steam has the least impact on the structure of *β*-Lg and on promoting the generation of glycation products. Superheated steam can be a suitable heating method for the *β*-Lg-glucose system. Circular dichroism spectrum shows a transition from *α*-helix to *β*-sheet/random coil. LC-MS/MS identified 10, 8, and 9 lysine rich glycation sites in SS, OB, and HA, respectively, and their DSP reflected the intensity of glycation reactions. Therefore, this study derived from a *β*-lactoglobulin-glucose model system, provides molecular-scale evidence on how different high-temperature heating modes regulate *β*-lactoglobulin glycation, providing a pathway for selecting gentler whey protein heat treatment in food manufacturing. However, it should be noted that the functional consequences of the observed structural changes and the safety–sensory profile of the glycated products have not been systematically evaluated. These aspects present critical objectives for future investigation.

## Figures and Tables

**Table 1:** Kinetic parameters for the glycation reaction under different treatments.

Treatment Methods	k (min^−1^)	[P]_eq_ (mg/mL)
SS	0.725 ± 0.211	0.303 ± 0.011
OB	1.520 ± 0.771	0.313 ± 0.010
HA	1.645 ± 0.336	0.294 ± 0.003

**Table 2:** The glycated peptides of glycated *β*-Lg under SS, OB, and HA conditions.

m/z	Start	End	Sequence	Modified Peptide	DSP (%)	Glycated Site
**SS**						
609.8422^+2^	1	11	(-)LIVTQTMKGLD(I)	690.8682^+2^	59.75 ± 0.36 ^b^	K8
565.8149^+2^	10	19	(G)LDIQKVAGTW(Y)	646.8403^+2^	42.61 ± 0.60 ^e^	K14
478.7627^+2^	33	41	(L)DAQSAPLRV(Y)	559.7880^+2^	21.10 ± 0.04 ^g^	R40
485.2668^+2^	46	54	(E)LKPTPEGDL(E)	566.2926^+2^	75.35 ± 0.12 ^a^	K47
745.3765^+2^	61	73	(K)WENGECAQKKIIA(E)	826.4034^+2^	44.56 ± 0.37 ^d^	K69/K70
495.7834^+2^	80	88	(P)AVFKIDALN(E)	576.811^+2^	50.30 ± 0.31 ^c^	K83
522.2728^+2^	122	130	(C)LVRTPEVDD(E)	603.2984^+2^	23.01 ± 0.17 ^f^	R124
551.6054^+3^	95	107	(V)LDTDYKKYLLFCM(E)	605.6245^+3^	9.33 ± 0.09 ^h^	K100/K101
**OB**						
755.7478^+3^	1	20	(-)LIVTQTMKGLDIQKVAGTWY(S)	809.7662^+3^	79.62 ± 0.01 ^b^	K8/K14
451.7588^+2^	12	19	(D)IQKVAGTW(Y)	532.7848^+2^	55.45 ± 0.47 ^d^	K14
421.2487^+2^	34	41	(D)AQSAPLRV(Y)	502.2746^+2^	8.71 ± 0.05 ^g^	R40
966.5033^+4^	15	50	(K)VAGTWYSLAMAASDISLLDAQSAPLRVYVEELKPTP(E)	1007.0171^+4^	62.13 ± 0.53 ^c^	R40/K47
745.3766^+2^	42	54	(V)YVEELKPTPEGDL(E)	826.4011^+2^	86.71 ± 0.02 ^a^	K47
810.0985^+3^	52	72	(E)GDLEILLQKWENGECAQKKII(A)	864.1193^+3^	51.47 ± 0.01 ^f^	K60/K69/K70
521.3194^+2^	87	95	(A)LNENKVLVL(D)	602.3452^+2^	54.78 ± 0.14 ^e^	K91
**HA**						
567.7965^+^	4	13	(V)TQTMKGLDIQ(K)	648.8212^+2^	87.71 ± 0.63 ^f^	K8
451.7593^+2^	12	19	(D)IQKVAGTW(Y)	532.7848^+2^	96.20 ± 0.01 ^a^	K14
535.3050^+2^	32	41	(L)LDAQSAPLRV(Y)	616.3301^+2^	45.60 ± 0.12 ^d^	R40
600.8233^+4^	42	61	(V)YVEELKPTPEGDLEILQKW(E)	641.3361^+4^	25.10 ± 0.41 ^e^	K60/K67
408.2171^+2^	123	129	(L)VRTPEVD(D)	489.2429^+2^	73.66 ± 0.25 ^b^	R124
641.8541^+4^	138	159	(D)KALKALPMHIRLSFNPTQLEEQ(C)	682.3691^+4^	54.87 ± 0.02 ^c^	K138/K141/R148

Letters a–h in the table mean significantly different (*p* < 0.05).

## Data Availability

The original contributions presented in the study are included in the article, further inquiries can be directed to the corresponding author.

## Abbreviations

The following abbreviations are used in this manuscript:

*β*-Lg	*β*-lactoglobulin
SS	superheated steam
HA	hot air
OB	oil bath
AGEs	advanced glycation end products
GC-MS	gas chromatography-mass spectrometry
CD	circular dichroism
UV	ultraviolet spectroscopy
SDS	sodium dodecyl sulfate
DOB	degree of browning
CML	carboxymethyllysine
5-HMF	5-hydroxymethylfurfural
F-AGEs	fluorescent AGEs
MLD	melanoidins
HPLC-HCD-MS/MS	high performance liquid chromatography-high collision dissociation-mass spectrometry/mass spectrometry
